# Expression of RFamide-Related Peptide-3 (RFRP-3) mRNA in Dorsomedial Hypothalamic Nucleus and KiSS-1 mRNA in Arcuate Nucleus of Rat during Pregnancy

**Published:** 2014-11-01

**Authors:** Fatemeh Sabet Sarvestani, Amin Tamadon, Omid Koohi-Hosseinabadi, Saeed Mohammadi Nezhad, Farhad Rahmanifar, Mohammad Reza Jafarzadeh Shirazi, Nader Tanideh, Ali Moghadam, Ali Niazi

**Affiliations:** 1 Transgenic Technology Research Center, Shiraz University of Medical Sciences, Shiraz, Iran; 2 Infertility Research Center, Shiraz University of Medical Sciences, Shiraz, Iran; 3 Laboratory Animal Center, Shiraz University of Medical Sciences, Shiraz, Iran; 4 Department of Basic Sciences, School of Veterinary Medicine, Shiraz University, Shiraz, Iran; 5 Department of Animal Sciences, School of Agriculture, Shiraz University, Shiraz, Iran; 6 Department of Pharmacology, School of Medicine, Shiraz University of Medical Sciences, Shiraz, Iran; 7 Biotechnology Institute, College of Agriculture, Shiraz University, Shiraz, Iran

**Keywords:** KiSS-1, RFamide-Related Peptide-3, Pregnancy, Dorsomedial Hypothalamic Nucleus, Arcuate Nucleus

## Abstract

**Background:**

RFamide-related peptide-3 (RFRP-3) and kisspeptin (KiSS-1) are known to respectively inhibit and stimulate gonadotropin releasing hormone (GnRH) and lute- inizing hormone (LH) secretion in rat. The aim of the present study was to evaluate the relative mRNA expression of RFRP-3 and KiSS-1 in the hypothalamus of pregnant rats.

**Materials and Methods:**

In a randomized controlled experimental study, the exact preg- nancy day of 18 Sprague-Dawley rats were confirmed using the vaginal smear method and were equally assigned to three groups of days 7, 14 and 21 of pregnancy. Four non- pregnant female rats were ovariectomized and assigned as the control group. All rats were decapitated, and the dorsomedial hypothalamic nucleus (DMH) and the arcuate nucleus (ARC) for detection of KiSS-1 mRNA were separated from their hypothalamus to detect RFRP-3 and KiSS-1 mRNA respectively. Then, their relative expressions were compared between control and pregnant groups using real-time polymerase chain reac- tion (PCR).

**Results:**

The relative expression of RFRP-3 mRNA in DMH did not change significantly during pregnancy (p>0.01). However, the relative expression of KiSS-1 mRNA in ARC was at its highest in day 7 of pregnancy and decreased until day 21 of pregnancy (p<0.01).

**Conclusion:**

Decrease in GnRH and LH secretion during the pregnancy of rat may be controlled by constant expression of RFRP-3 mRNA and reduced expression of KiSS-1 mRNA in hypothalamus.

## Introduction

There is no follicular development during pregnancy in the rat compared to the changes during the estrous cycle (1). During pregnancy, it has been documented that serum luteinizing hormone (LH) levels tend to decrease, reaching its lowest at mid-pregnancy and tend to recover by the end of gestation (2, 3). During the first 11 days of pregnancy in rat, serum LH concentration was shown to be higher than the period between days 13-19. A progressive increase then occurs beginning on day 20 and continuing to term, but is not contiguous with the postpartum ovulation inducing surge of LH (3). Kisspeptin and RFamide-related peptide-3 (RFRP-3) were recognized as regulators of gonadotropin releasing hormone (GnRH) and LH secretion in several species, including the rat (4).

Kisspeptins belong to a family of peptides which are encoded by the *KiSS-1*for 20 minutes and the gene and are natural ligands of the G protein-coupled receptor 54 (GPR54). Kisspeptin has a fundamental role in the control of gonadal axis (5, 6). It has been shown that kisspeptin neurons stimulate LH release by affecting GnRH neurons (7). This is based on the observation that the excitatory effect of kisspeptin on gonadotropin secretion was inhibited by GnRH antagonists (8). Almost all GnRH neurons express GPR54 (9) and many kisspeptin neurons in rats express estradiol receptor á (ERá) (10). Thus, it is possible that estrogen effects on GnRH neurons are mediated through these cells. KiSS-1 mRNA and encoded peptide were detected in the arcuate nucleus (ARC) and anteroventral periventricular nucleus (AVPV) of rodents using immunohistochemistry and in situ hybridization (11, 12). Kisspeptin neurons of the ARC might be the GnRH pulse generating center while AVPV might have a role in preovulatory GnRH/LH surge (13). KiSS-1 mRNA levels in AVPV was highest during the proestrus and lowest during metestrus (14). Furthermore, the level of KiSS-1 mRNA in ARC was highest during diestrus and lowest during proestrus (14), milking (15, 16) and malnutrition condition (17).

Gonadotropin-inhibitory hormone (GnIH) is a novel hypothalamic neuropeptide was discovered in birds as an inhibitory factor for gonadotropin release (18). RFRP-3 is a mammalian GnIH ortholog that inhibits gonadotropin synthesis and release in mammals through actions on GnRH neurons and gonadotropes, mediated via the GnIH receptor (GnIH-R), GPR147 (19). This peptide, was identified in the brain of rodents, modulates the negative feedback effect of estrogen on gonadotropin secretion (20). The RFRP-ir cells, clustering in the dorsomedial nucleus of the hypothalamus (DMH), were identified in hamsters, rats and mice (20-22). Inhibitory effects of RFRP on pituitary gonadotropins decreases reproductive activity of male and female rats (23, 24), and sheep (25, 26). Axons of RFRP neurons are projected to GnRH neurons in rodents (20) and RFRP-3 peptide has an inhibitory effect on GnRH neurons in mouse (27) and rat (28). Moreover, RFRP cells in hamster (20) and mouse (27) express ERá and administration of estradiol 17β can dramatically decrease prepro-RFRP mRNA in ovariectomized rats (27).

The aim of the present study was to evaluate the relative expression of RFRP-3 and KiSS-1 mRNAs in the hypothalamus of pregnant rats on days 7, 14 and 21 after mating. To achieve this goal, we need to determine pregnancy earlier than day 7 after mating with high accuracy and without using hormonal estrous synchronization. Hitherto, different non-invasive methods such as vaginal plug observation (day 1 after mating) (29), ultrasonography (day 8 after mating) (30), observation of abdominal distention and fetal palpation (day 13 after mating) (31) were presented for pregnancy detection in rat. For the first time, we present a novel low-cost and noninvasive method to increase the chances of making rats pregnant on day 5 after mating, and to determine the exact time of their pregnancy.

## Materials and Methods

### Study 1: early pregnancy detection

In a randomized controlled experimental study, 40 mature female Sprague-Dawley rats (body weight 150-250 g) were selected and housed in The Laboratory Animal Center of Shiraz University of Medical Sciences, Shiraz, Iran. The animals were housed in standard cages, six per cage, in a controlled temperature room (22℃), with a 12 hours light and 12 hours dark cycle. Standard laboratory chow and tap water were available ad libitum.

Phases of their estrous cycle were determined by microscope observation of their vaginal smears (32). The rats at the proestrous or estrous stage were transferred to the cage of mature male rats (body weight 250-350 g) with 3:1 ratio and left overnight. The presence of vaginal plug was recorded the next morning and female rats were separated from the males. On days 4 and 5 after estrus, vaginal smear was evaluated once again and their cellular characteristics were determined under light microscope. Finally, all females were checked for pregnancy. Abdomen enlargement of female rats on day 16 after mating and/or post-parturition observation of their litters were considered as positive pregnancy. All the above was repeated three times.

The rats were assigned into three groups. The first group was the rats with diestrous cell characteristics in day 4 and metestrous/diestrous cell characteristics in day 5. The second group was the rats with metestrous cell characteristics in day 4 and metestrous/diestrous cell characteristics in day 5. The other cell characteristics in days 4 and 5 were assigned to the third group.

### Study 2: expression of RFRP-3 and KiSS-1 mRNA in hypothalamus

#### Animals, experimental groups, and sampling

Twenty two adult (3-4 months old) female Sprague-Dawley rats (*Rattus norvegicus*) weighing between 170 and 220 g were used in the present randomized controlled experimental study. The rats were randomly selected and housed in The Laboratory Animal Center of Shiraz University of Medical Sciences, Shiraz, Iran under controlled temperature (22℃) and lighting (12:12 light to dark ratio; light on at 7:30 AM) conditions. The rats were housed in compliance with the recommendations of The Animal Care Committee of the Shiraz University of Medical Sciences. All experimental procedures were carried out between 12.00-2.00 PM. The exact pregnancy day of the 18 rats was confirmed using the vaginal smear method (study 1). The rats were then randomly assigned in three equal groups of 7, 14 and 21 days of pregnancy (n=6).

Four ovariectomized rats, selected randomly, were used as the control group. The rats were anesthetized by an intraperitoneal injection of ketamine (100 mg/kg, Woerden, Netherlands) and xylazine (7 mg/kg, Alfazyne, Woerden, Netherlands) and ovariectomized through ventral midline incision. Further procedures were carried out after a 2-week recovery period.

The pregnant and ovariectomized rats were decapitated and brains were removed immediately. Pregnancy of 18 rats was confirmed with certainty by observing their pregnant uterus. The diencephalon was dissected out by an anterior coronal section, anterior to the optic chiasm, and a posterior coronal cut at the posterior border of the mammillary bodies. To separate ARC from AVPV, a third coronal cut was made through the middle of the optic tract, just rostral to infundibulum (33). The specimens consisting of ARC and DMH were stored in liquid nitrogen until further analysis.

### Real-time polymerase chain reaction (PCR)

Total RNA was extracted, using the RNX-Plus buffer (Cinnagen, Tehran, Iran). Briefly, the tissue (100 mg) was ground in liquid nitrogen, transferred to RNX-Plus buffer (1 mL) in an RNase-free microtube, mixed thoroughly, and kept at room temperature for 5 minutes. Chloroform (0.2 mL) was added to the slurry and mixed gently. The mixture was centrifuged at 12,000 ×g (4℃) for 20 minutes and the supernatant was transferred to another tube and precipitated with an equal volume of isopropanol for 15 minutes. The RNA pellet was washed with 75% ethanol and quickly dried and re-suspended in 50 µL RNase-free water. The purified total RNA was quantified by Nano-Drop ND 1000 spectrophotometer (Nano-Drop Technologies, Wilmington, DE, USA). The DNase treatment was carried out using the DNase kit (Fermentas, St. Leon-Roth, Germany) according to the manufacturer¡¯s instructions. The DNase-treated RNA (3 µg) was used for the first strand cDNA synthesis, using 100 pmol oligo-dT, 15 pmol dNTPs, 20 U RNase inhibitor, and 200 U M-Mulv reverse transcriptase (Fermentas, St. Leon-Roth, Germany) in a 20 µL final volume. Primers were designed using Allele ID 7 software (Premier Biosoft International, Palo Alto, USA) for the reference gene, KiSS-1 (NM_181692) and RFRP-3 (NM_023952). The rat *glyceraldehyde-3-phosphate dehydrogenase* (*GAPDH*) gene (M32599) was used as the reference gene for data normalization (Table 1). Relative real-time PCR was performed in a 20 µL volume containing 1 µL cDNA, 1X Syber Green buffer and 4 pmol of each primer. The amplification reactions were carried out in a Line-Gene K thermal cycler (BIOER Technology Co., Ltd, Hangzhou, China) under the following conditions: 2 minutes at 94℃, 40 cycles of 94℃ for 10 seconds, 57℃ for 15 seconds, and 72℃ for 30 seconds. After 40 cycles, the specificity of the amplifications was tested by analyzing melting curves with the temperature ranging from 50℃ to 95℃. All amplification reactions were repeated 3 times under identical conditions, including a negative control and 5 standard samples. To ensure that the PCR products were generated from cDNA, but not the genomic DNA, proper control reactions were implemented in the absence of reverse transcriptase. For quantitative real-time PCR data, the relative expression of KiSS-1 was calculated based on the threshold cycle (C_T_) method. The CT for each sample was calculated, using Line-gene K software (34). Accordingly, the fold expression of the target mRNAs over the reference values was calculated by the equation 2-_ΔΔCT_ (35), where ΔC_T_ is determined by subtracting the corresponding *GAPDH* C_T_ value (internal control) from the specific C_T_ of the target (KiSS-1 or RFRP-3). The ΔΔC_T_ was obtained by subtracting the ΔC_T_ of each experimental sample from that of the control (ovariectomized rats).

**Table 1:** Sequences of real time polymerase chain reaction (PCR) primers for evaluation of the relative expression of RFRP-3 and KiSS1 genes in rat


Primer	Sequence	Amplicon length (bp)

**KiSS1-F**	TGCTGCTTCTCCTCTGTG	116
**KiSS1-R**	CCAGGCATTAACGAGTTCC	
**RFRP-3-F**	CTCAGCAGCCAACCTTCC	165
**RFRP-3-R**	AAACCAGCCAGTGTCTTG	
**GAPDH-F**	AAGAAGGTGGTGAAGCAGGCATC	112
**GAPDH-R**	CGAAGGTGGAAGAGTGGGAGTTG	


KiSS1; Kisspeptin, RFRP-3; RFamide-related peptide-3 and GAPDH; Glyceraldehyde-3-phosphate dehydrogenase.

### Statistical analysis

In study 1, relationship between the cell characteristics of vaginal smear (proestrus, estrus, metestrus and diestrus) on days 4 and 5 after mating and positive and negative results of pregnancy were compared using the Chi-square test (SPSS for Windows, version 11.5, SPSS Inc, Chicago, Illinois). Percent of pregnant rats between three groups were analyzed using one-way analysis of variance (ANOVA) (SAS 9.1 SAS Institute Inc., Cary, NC). Tukey post hoc test was used for comparison of means within the groups. Values of p≤0.05 were considered significant.

In study 2, the data on relative expression of KiSS-1 and RFRP-3 genes were subjected to the test of normality and analyzed by one-way ANOVA, and mean separation was performed by Tukey’s test at p=0.01. Group means and their standard errors are reported in the text and figures (GraphPad Prism v 5.01, GraphPad software Inc., San Diego, CA, USA).

## Results

**Fig 1:**
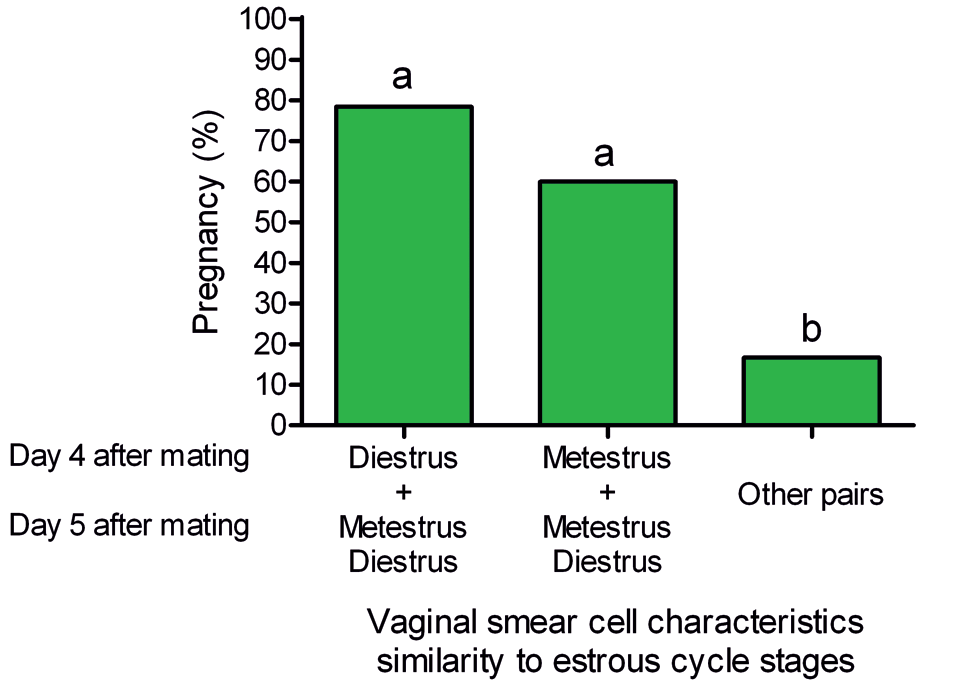
Accuracy of rat pregnancy detection by vaginal smear evaluation on days 4 and 5 after mating. a, b; Bars labeled with different letters are significantly different from each other at p<0.05.

In study 1, there was a positive association between pregnancy of rats and vaginal smear cell characteristics of metestrus (the same proportion among leukocytes, cornified and nucleated epithelial cells) or diestrous (a predominance of leukocytes) stages observed in days 4 and 5 after mating (p=0.001). If diestrous cell characteristics were observed in vaginal smear on day 4 after mating, and metestrous or diestrous cell characteristics were detected on day 5 after mating, 78.4% of rats would be pregnant. The cell characteristics of vaginal smear on days 4 and 5 were compared with cell characteristics of vaginal smear in different stages of estrous cycle in rat is shown in fig 1. Moreover, if metestrous cell characteristics were observed on day 4 after mating and metestrous or diestrous cell characteristics were detected on day 5 after mating, accuracy of pregnancy detection dropped to 60% which was not significantly different with the previous case (diestrous on day 4 and metestrous/diestrous on day 5). Only 16.7% of pregnant rats showed other cases of cellular characteristics of estrous cycle on days 4 and 5 after mating with chances to be pregnant was less than the two previous cases (p<0.05).

**Fig 2:**
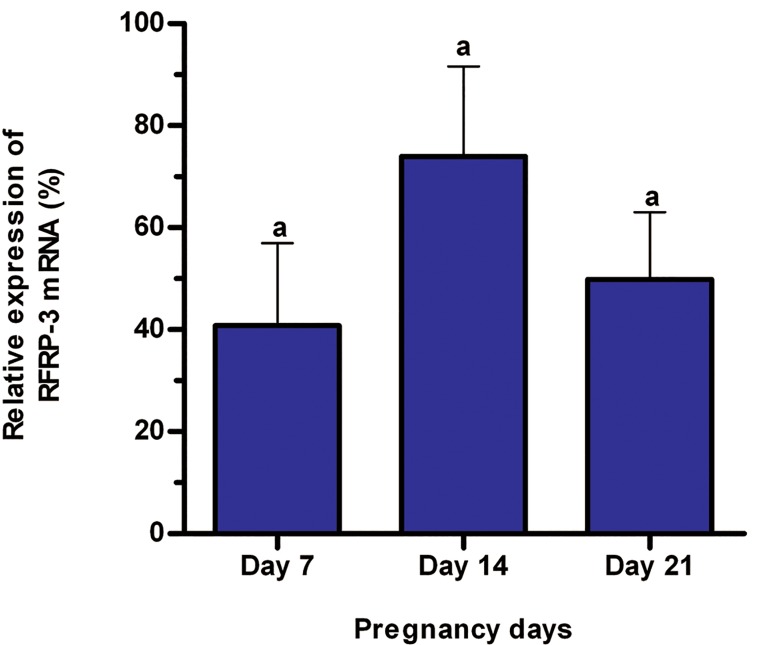
In study 2, the mean and standard error of relative expression of RFRP-3 mRNA in DMH did not change during pregnancy (p>0.01, Fig 2). However, the relative expression of KiSS-1 mRNA in ARC was at its highest on day 7 of pregnancy and decreased until day 21 of pregnancy (p<0.01, Fig 3).

**Fig 3:**
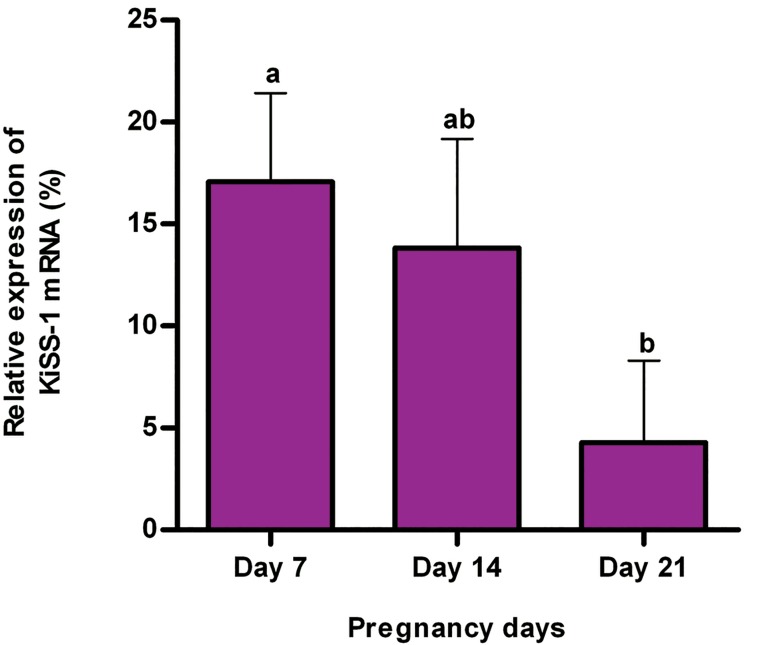
Effect of pregnancy on the relative expression of KiSS-1 gene (mean ± SE) in the hypothalamic arcuate nucleus (ARC) of rats (n=6 for each pregnancy day). a, b; Different letters indicate significant difference (p<0.01).

In study 2, the mean and standard error of relative expression of RFRP-3 mRNA in DMH did not change during pregnancy (p>0.01, Fig 2). However, the relative expression of KiSS-1 mRNA in ARC was at its highest on day 7 of pregnancy and decreased until day 21 of pregnancy (p<0.01, Fig 3).

## Discussion

The relative expression of KiSS-1 mRNA in ARC was at its peak in the first week of pregnancy and decreased 4-fold in the third week of pregnancy in rat. In contrast to our results, Roa et al. (36) reported that KiSS-1 mRNA increased during the pregnancy in rat brains. In humans, kisspeptin levels increased by 940-fold in the first trimester in comparison with non-pregnant woman and further increased to some 7000-fold higher in the third trimester (37). Level of expression of KiSS-1 mRNA is higher in the first trimester placenta than in term placenta in humans (38), apparently contrasting with higher circulating kisspeptin levels reported during pregnancy (37). Considering the ability of systemically delivered KiSS-1 peptides to release LH (8, 39-42), this phenomenon seems to be at odds with the reported increase in serum kisspeptin concentrations in human pregnancy (37). Although human and rat differ with regard to length of gestation and placental structure, the spatial and temporal expression of KiSS-1 mRNA are similar. Kisspeptin and its receptors are detected in rat trophoblast (43). In specific, KiSS-1 mRNA is expressed in the trophoblast giant cells of the rodent placenta (44), which are responsible for early invasion of spiral arteries and replacement of the endovasculature. These cells have the same functional phenotype as the human extravillous trophoblasts. As in humans, levels of KISS-1 and its receptor gradually decline during placental maturation and are not detectable at embryonic day 18.5 (44). The finding of the highest expression level of KISS-1 mRNA in trophoblast cells during the first trimester in humans and at day 12.5 of pregnancy in rodents and decrease in day 15.5 to no detectable expression on day 18.5 of pregnancy in rat coincides with the time of peak trophoblast invasion when regulation of this process is of critical importance (45, 46).

It has been shown that the number of GnIH neurons have a positive correlation with plasma progesterone concentration (47) and that GnIH neurons are regulated by progesterone (48). Responsiveness to RFRP-3 mRNA at pregnancy may derive from the combined exposure to high levels of progesterone and suppression of LH levels during pregnancy in rat (49). On the other hand, total cortisol and progesterone increased significantly from one trimester of pregnancy to the next in humans (50). Increase of glucocorticoids caused an increase in RFRP that contributes to hypothalamic suppression of reproductive function in rat (51).

## Conclusion

Decrease of GnRH and LH secretion during rat pregnancy may be controlled by constant expression of RFRP-3 mRNA and reduced expression of KiSS-1 mRNA. On the other hand, methods of early detection of pregnancy and exact determination of pregnancy time in rats are widely applied on pregnant rats. Our presented method, in addition to increasing the probability of rat pregnancy after the first mating event, can detect pregnancy with an accuracy of more than 60% on day 5.
